# Supplementary material for: Brain inspired neuronal silencing mechanism to enable reliable sequence identification
Source: Sci Rep. 2022 Sep 29;12:16003. doi: 10.1038/s41598-022-20337-x (PMC9523036; doi:10.1038/s41598-022-20337-x)
Supplement: Supplementary file 1 — Supplementary Figures. [file 41598_2022_20337_MOESM1_ESM.pdf]

Supplementary Information

Brain inspired neuronal silencing mechanism to enable reliable sequence identification

Shiri Hodassman[1†], Yuval Meir[1†], Karin Kisos[1], Itamar Ben-Noam[1], Yael Tugendhaft[1], Amir Goldental[1], Roni Vardi[2†] & Ido Kanter[1,2*]

[1]Department of Physics, Bar-Ilan University; Ramat-Gan, 52900, Israel.

[2]Gonda Interdisciplinary Brain Research Center, Bar-Ilan University; Ramat-Gan, 52900, Israel.

*Corresponding author. Email: ido.kanter@biu.ac.il

†These authors contributed equally to this work.

This PDF file includes:

Figures. S1 to S6

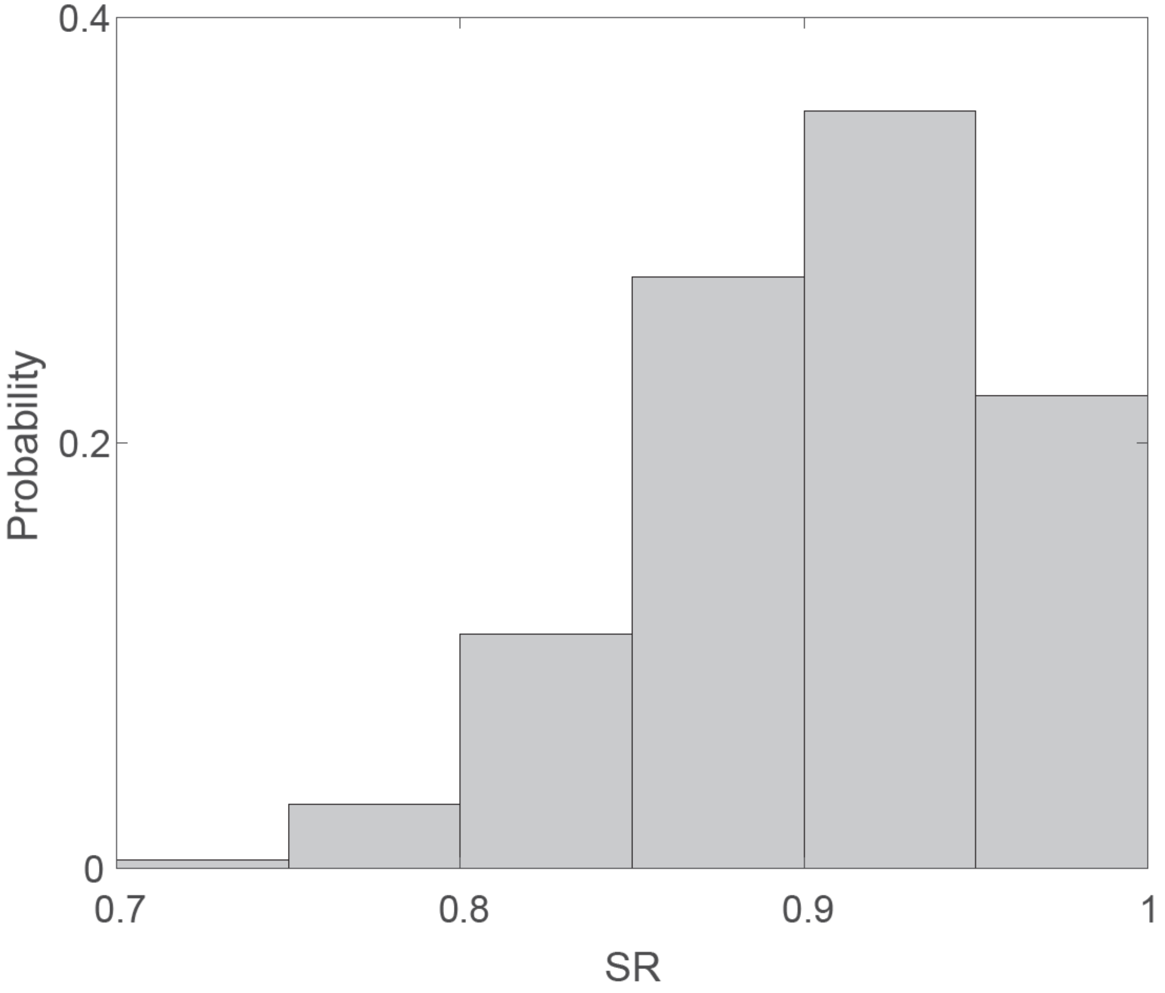

Supplementary Figure 1. SRs of the ID-net for one digit without silenced nodes. Histogram of the SRs of the ID-net (Fig. 1b) for individual digits without silenced nodes using 1000 training examples per digit, where $\eta = 7.5 \cdot 10^{-5}$, $\alpha = 1 \cdot 10^{-7}$, and $\mu = 0.9$ (see Methods). The histogram includes the estimated SR for each digit using 1000 test examples, where the measure is repeated 100 times. The averaged SR is ~0.90.

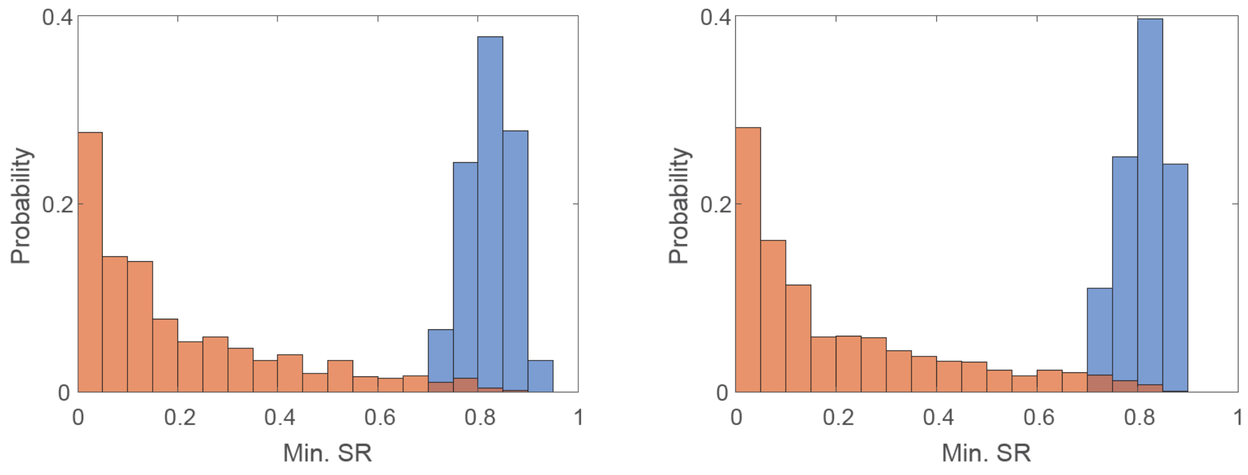

Supplementary Figure 2. Robustness of ID-net with multiple trained sequences. The ID-net (Fig. 1b) trained using 1000 training sequences for each embedded sequence, each of which consists of 10 MNIST digits with $d = 30$ and $d_1 = 20$ frames (Fig. 1c) separated by $10\ ms$ with consecutive digits separated by $\Delta t = 200\ ms$. **Left:** Similar to the right panel of Fig. 2a with three trained sequences. There is a small overlap between the orange and the blue histograms, however, a threshold 0.73 predicts correctly with probability ~0.9773 if the order of the input sequence is one of the correct sequences or a wrong one. **Right:** Similar to the left panel of Fig. 1a with four trained sequences. The overlap between the orange and the blue histograms increases, however a threshold of 0.71 predicts correctly with probability ~0.97 if the order of the input sequence is one of the correct sequences or wrong. The mutual order of the multiple embedded sequences is very similar. Each pair of the embedded multiple sequences differs in swapping of one or two pairs of digits only.

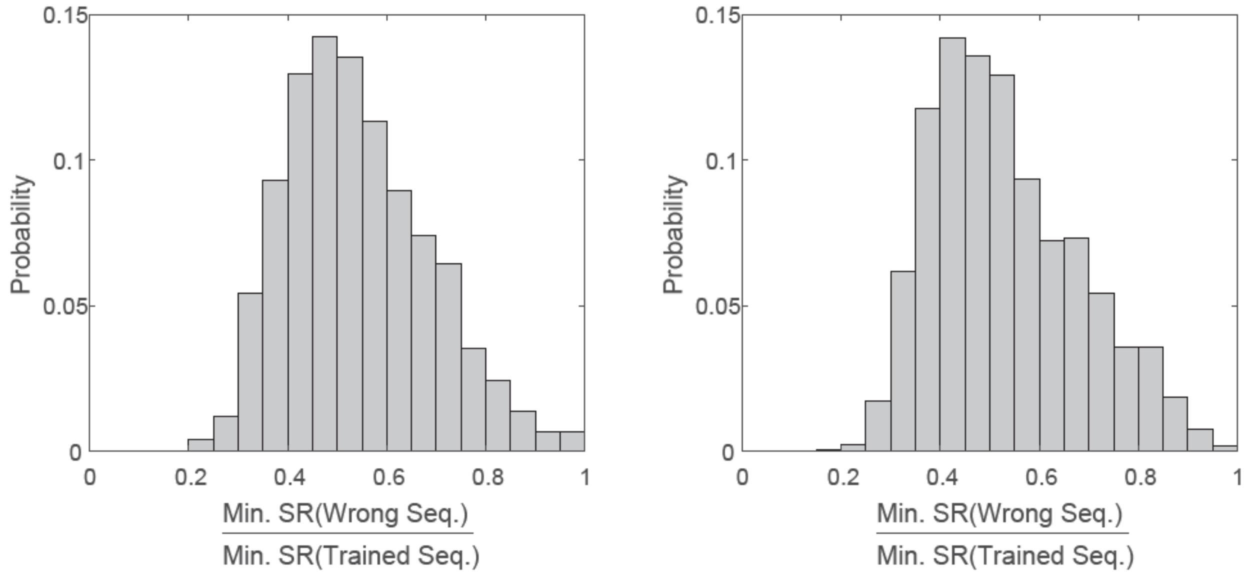

Supplementary Figure 3. A fitted threshold for each trained sequence enhances the gap. Histograms for the ratio between the minimal SR for a wrong sequence (Fig. 1e) and for the trained one. **Left:** Histogram for a trained sequence, where the data is taken from the right panel of Fig. 2a. **Right:** Histogram for two trained sequences, where the data is taken from the right panel of Fig. 2b. Since the ratio is less than 1, a fitted threshold for each trained sequence can be used to better distinguish between the embedded sequences and sequences with the one of the three imperfections (Fig. 1e).

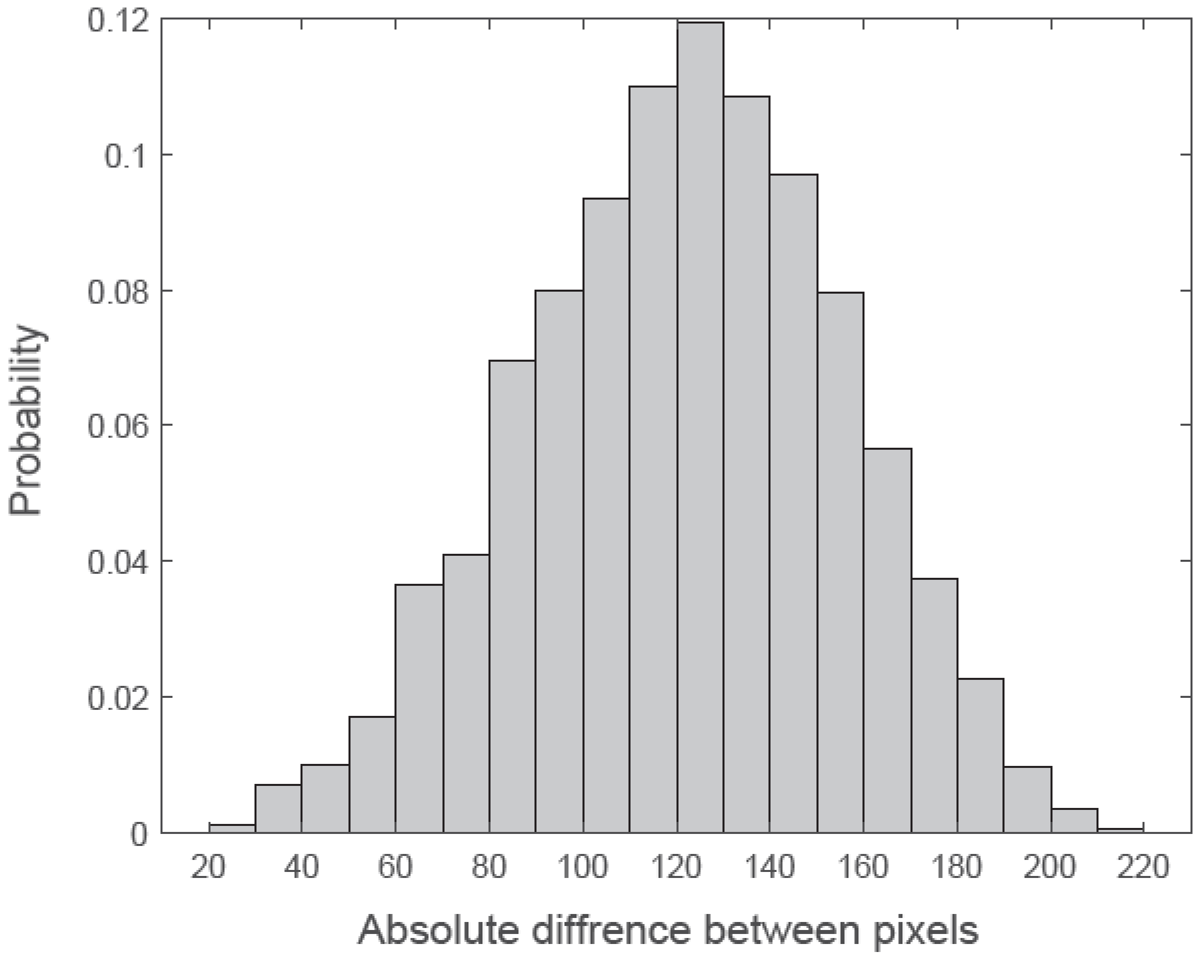

Supplementary Figure 4. Histogram of the absolute difference between gray-level pixels greater than 100 of two MNIST examples with the same label. The histogram is constructed from 100 randomly selected pairs for each one of the 10 labels. For each pair, the differences between pixels with gray-level greater than 100 in the first example and the corresponding pixels in the second examples within the pair are calculated. The histogram is built from 1000 pairs, 100 for each label. Note that the minimal difference between two handwritings is greater than 20, where the maximal difference between two digits of the same handwriting is 20, i.e. the average difference is 10 (Fig. 3).

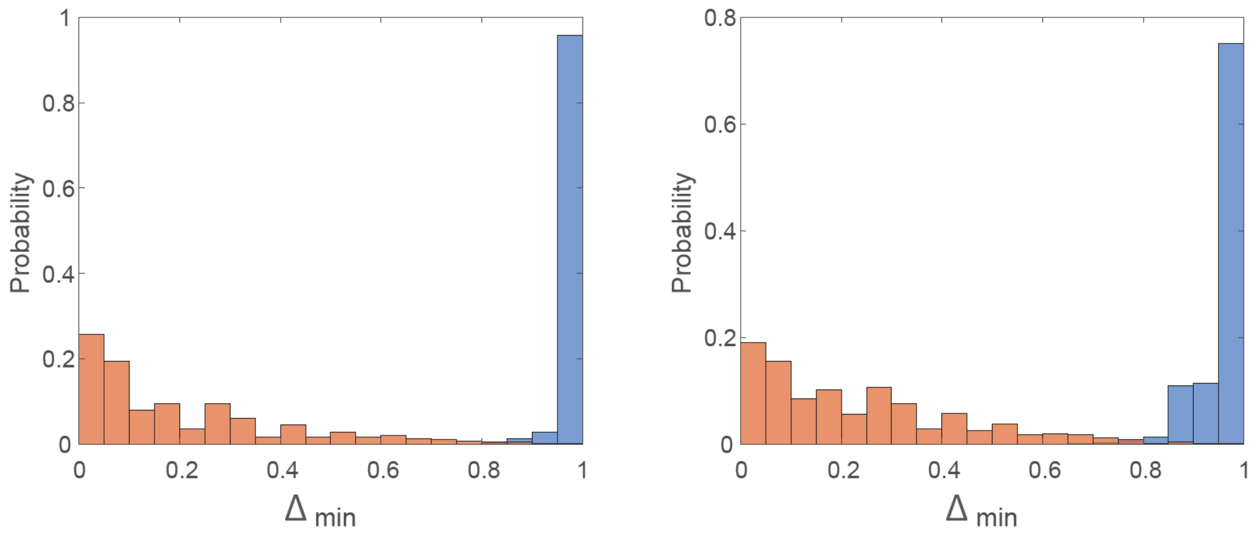

Supplementary Figure 5. The capability of ID-net to identify several writer-dependent sequences. The ID-net (Fig. 1b) is trained on several individual's handwritings. The training of each handwriting consists of a sequence of 10 different MNIST digits, with 50 additional similar synthetic sequences generated by adding integer noise in the range [-20, 20] to pixels with a gray-level greater than 100. The 10 digits are ordered differently, such that in the same position along the sequence different digits appear for different embedded sequences. For each predicted test digit, the gap between the highest and next highest firing output nodes is normalized by d_1 to the range $[0, 1]$, and its minimal value among the 10 digits, Δ_{min}, is selected. The used parameter are: $\eta = 3.3 \cdot 10^{-3}; \alpha = 1.6 \cdot 10^{-5}$.

Left panel: Similar to Fig. 3c with two embedded writer-dependent sequences. A threshold of 0.80 results in a probability of ~0.994 for correct classification of an input as one of the embedded sequences. Note, that we assume a possible opponent has the knowledge of the individual handwriting, the speed of the sequence, as well as the correct position of eight digits among the ten.

Right panel: Similar to Fig. 3c with three embedded writer-dependent sequences. A threshold of 0.76 results in accuracy of ~0.985, where accuracy is defined as normalized true-positive plus true-negative.

The left histogram is averaged over $1440 \cdot 2$ instances composed of 40 samples and the right histogram over $1440 \cdot 3$ instances composed of 40 samples.

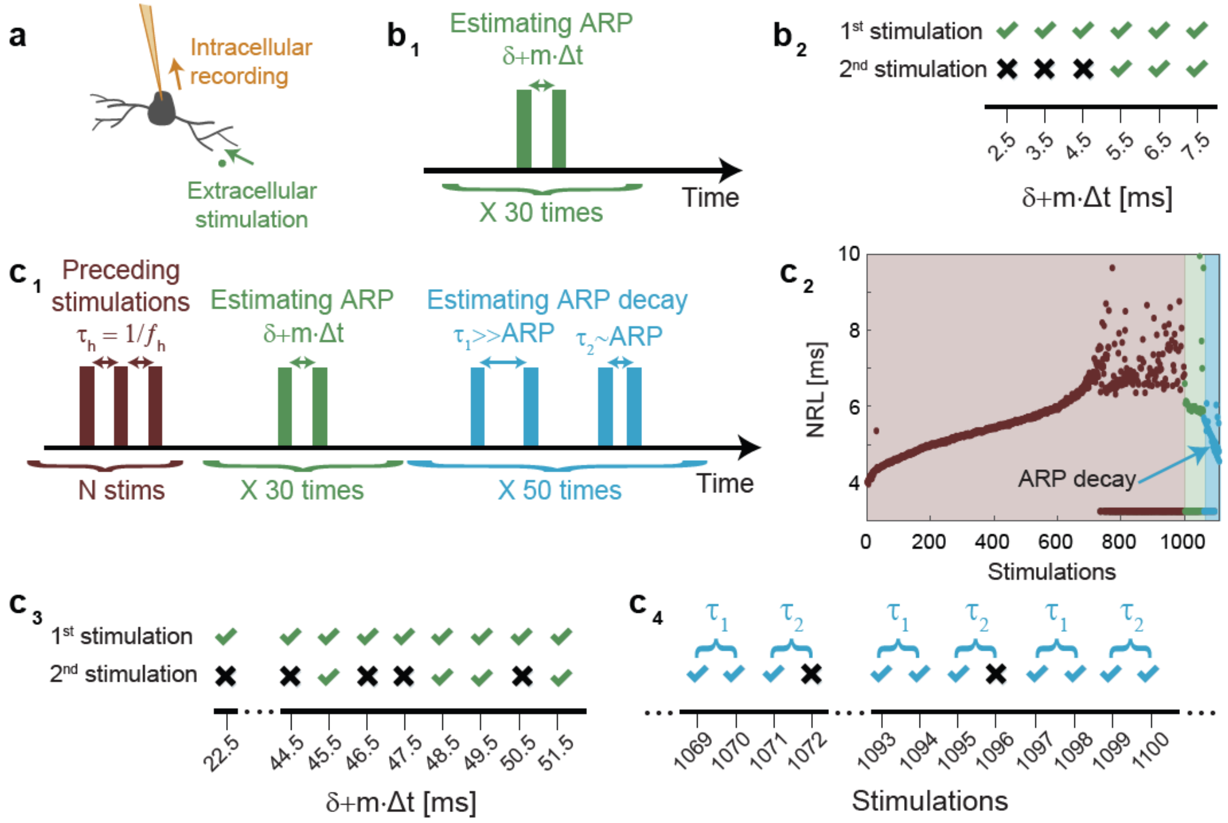

Supplementary Figure 6. The relaxation period of the absolute refractory period. (**a**) Scheme of an in-vitro neuron in a synaptic blocked culture that is stimulated via one of its dendrites and recorded intracellularly. (**b1**) Scheme of the experiment measuring the absolute refractory period (ARP) using pairs of stimulation scheduling, with increasing intra-pair time-lags. (**b2**) Neuronal responses for stimulation scheduling at 1 Hz with δ = 2.5 ms, Δt = 1 ms in **b1** (V = evoked spike; X = no evoked spike), indicating a 5.5 ms resting ARP. **c** The ARP is a reversible process that after sufficient time returns to its initial value, e.g., 5.5 ms resting ARP (**b2**). The estimation of the decay time of the ARP requires a new type of experiment implementing the following three steps in one stimulation schedule. (**c1**) Scheme of stimulation scheduling that consists of the following three steps: 1000 preceding stimulations (at f_h=25 Hz), leading to the intermittent phase (brown); 50 pairs of stimulations (at 5 Hz), with increasing intra time-lags (green) to estimate the stretched ARP (δ = 22.5 ms, Δt = 1 ms); and alternating pairs of stimulations (at 0.5 Hz) (blue): one with $\tau_1 = 17\, ms \gg resting\ ARP$ and one with $\tau_2 = 6.5\, ms \sim resting\ ARP$. (**c2**) Neuronal response latency (NRL) and response failures

(bottom) for the stimulation scheduling $\mathbf{c_1}$ of neuron $\mathbf{b_2}$. ($\mathbf{c_3}$) Stretched ARP = 45.5 ms as measured by the green pairs $\mathbf{c_1}$. The elongation of the ARP from 5.5 ms to 45.5 ms is an example where the response probability of a neuron is reduced to 50% since the second stimulation within each pair results in response failure. ($\mathbf{c_4}$) Responses of the alternating pairs (blue in $\mathbf{c_1}$), where responses for the τ_2 pairs start at stimulation 1100, which occurs a minute after the termination of the preceding stimulations (brown in $\mathbf{c_1}$); the NRL nearly decays to its resting value (blue in $\mathbf{c_2}$).

Results indicate a decay of the stretched ARP back to resting ARP after approximately one minute $\mathbf{c_4}$. This relaxation is correlated with the decay of the NRL back to its initial value ($\mathbf{c_2}$), as confirmed by examining different neurons (n = 11, mean = 61.5 s, STD = 30.3 s).
